# Integrated care in German mental health services as benefit for relatives – a qualitative study

**DOI:** 10.1186/s12888-016-0760-6

**Published:** 2016-02-27

**Authors:** Jan Valentini, Daniel Ruppert, Julia Magez, Constance Stegbauer, Anke Bramesfeld, Katja Goetz

**Affiliations:** Department of General Practice and Health Services Research, University Hospital Heidelberg, Voßstr. 2, 69115 Heidelberg, Germany; AQUA – Institute for Applied Quality Improvement and Research in Health Care, Maschmühlenweg 8-10, 37073 Göttingen, Germany; Institute of Family Medicine, University Hospital Schleswig-Holstein, Campus Luebeck, Ratzeburger Allee 160, 23538 Luebeck, Germany

**Keywords:** Integrated care, Mental health services, Health services research, Qualitative research, Relatives

## Abstract

**Background:**

As mental health services undergo the process of deinstitutionalization, this is resulting in a higher burden of care for relatives. Evidence suggests that interventions for carers have a beneficial impact on their psychological health. A reduction of responsibility for relatives is linked with a significantly improved outcome for the severely mentally ill. The aim of the study was to explore the relatives’ experiences with severely mentally ill patients in different integrated care service providers.

**Methods:**

Semi-structured focus groups and interviews were conducted with 24 relatives of patients receiving community based integrated care for severe mental illness. The collected data was transcribed and evaluated using qualitative content analysis. A deductive-inductive approach was used in generating thematic categories.

**Results:**

Four main categories were found related to the structural aspects of the integrated care services and for the experiences of the relatives within these services. Relatives reported that the services offered significant relief and substantial support in daily life. In addition, relatives felt a reduced burden of carer responsibility and therefore that they were provided with more protection and stability. This resulted in a sense of encouragement and not feeling left alone to face challenges.

**Conclusion:**

Relatives are a critical resource for patients suffering from mental health problems and benefit from formal structures and interventions to support them in carer role. An important need is to ensure continuity of care for patients and the bridging of gaps concerning information and support needs for relatives when providing integrated mental health services in the community.

## Background

The treatment of severe mental illness is in the process of deinstitutionalization, which is resulting in a higher burden of care for relatives [1, 2]. In addition, it is critical to ensure that a sufficient care and oversight is provided to ensure safe treatment of the severely mentally ill in community settings [3]. Due to the additional burden of care, relatives may neglect their own needs, which in turn have a negative impact on their own mental health.

Several reviews have shown that carer-based interventions have a beneficial impact on relatives by enhancing their psychological health which leads to a reduction in the burden of care [4–7]. A reduction of responsibility for relatives is associated with a significantly improved outcome for severely mentally ill [8]. Various studies recommend that interventions focused on supporting relatives in their caring role should be integrated in mental health services [9, 10].

For more than three decades, international evidence has been building, which shows that the treatment and care of mentally ill persons in a community care setting results in better outcomes [8, 11]. Models such as integrated care (IC) and assertive community treatment (ACT) are examples of best practice community care in the treatment of mental disorders [12–14].

IC is a funding model to facilitate the provision of mental health services for patients in community settings. The IC model for severely mentally ill persons is also gaining in importance in Germany [15–17]. In 2009, one of the largest national statutory health insurance company the “Techniker Krankenkasse” (TK) introduced an IC model called “NetzWerk psychische Gesundheit” (NWpG) and it has been implemented by several mental health service providers across Germany. The structural components of the service providers included home treatment, 24-h accessibility, case management as well as a crisis intervention apartment to avoid hospitalization and finally information supply. In addition, relational therapists are integrated into the health professional team and differ from the other staff as they are the individual contact person for each specific patient. This is with the intent to establish continuity in the therapeutic relationship. Beside these core structural elements, service providers freely determine the form of cooperation with other services and the manner of organization within their service [18, 19].

Some qualitative studies focused on the role of relatives caring for patients with severe mental health problems [20–23]. They found that relatives experience a strong and demanding responsibility which has a far reaching influence in carers’ daily life. Physical, emotional, social and financial aspects are often affected and to a great extend needs of relatives are not satisfactory coped by the current mental health system. Therefore, community based care have a key role in supporting the relatives and is internationally becoming increasingly important. Some publications showed that carer-based interventions have a positive impact on relatives and reduce their sense of burden. This may lead to improved outcomes for patients with severe mental illness [9, 24–27].

The aim of the current study was to investigate the experiences of relatives caring for severely mentally ill patients in the NWpG. An understanding of these experiences would enable the identification of fundamental needs and requirements of relatives, in order to improve quality of care for the family constellation when impacted by severe mental illness. Results may not only apply to the German health care system but may be transferred on different mental health delivery systems [1, 4, 28].

## Methods

### Study design

The present study was designed as a qualitative study. Focus groups and additional interviews were performed based on best practice guidelines for qualitative studies [29]. Individual interviews took place with those participants who could not or who preferred to take part in the focus group interviews. This was not pre-planned as an additional source of data collection, and the analysis did not seek to compare (i.e., triangulate) the findings of the focus group and individual interviews but instead analyzed them as a combined data set. The qualitative study design was chosen to allow an intensive analysis of subjective motives, attitudes and needs of participants. Qualitative methods can supply a greater depth of information about particular research questions and permit the generation of hypotheses for further research with quantitative methods. Semi- standardized, guideline-based focus groups were carried out with a convenience sample of patient’s relatives. The interaction between the participants in a group context allowed for synergistic gains in that critical examination of the opinions and statements expressed during the discussion could be further drawn out by the interviewer. This leads to a deep and broad insight into the examined topics, which was more detailed than only the opinion of individuals via one-on-one interviews [30].

### Sample data and recruitment

The participants in this study were relatives of patients with severe mental illness who were receiving community-based care within the NWpG [18]. Five out of the currently 17 existing service providers within the integrated care model NWpG were targeted for this study. They were selected on the basis of a significant variation in structural aspects and processes within their service as well as in patient-related outcomes. Table 1 gives an overview of the selected service providers. They were located across Germany in attempt to limit possible regional or cultural selection biases.

**Table 1:** Characteristics of the selected service provider sites

Service provider site	Compass direction	Number of patients^a^	Proportion of NWpG patients^a^	Proportion of Home treatment utilization^a^
1	Central	395	74 %	3 %
2	West	70	77 %	14 %
3	East	1400	85 %	46 %
4	North	5487	32 %	55 %
5	South	454	28 %	0 %

NWpG NetzWerk psychische Gesundheit; ^a^Data from 2013

The 5 chosen service providers assisted in recruitment of participant for the focus groups. A convenience sample of relatives was chosen. Relatives were addressed directly by the service providers’ staff and information containing background, procedure and goals of the study as well as an informed consent with references to the privacy policy of the study was handed out to interested relatives. The participation was voluntary. Study participants signed an informed consent form in advance of participation and received a financial incentive of 50 Euros. Focus groups were performed at three sites. Group size varied from six to eight people per focus group. In two service providers the number of relatives recruited was not sufficient to conduct a focus group; therefore two phone-interviews and one personal interview (based on the interview guide used in the focus groups) were performed. An overview of participants regarding each service provider site is given in Table 2. An integration of focus groups and individual interview data was obtained. The combination of individual interviews and focus groups had the potential for identification of individual and contextual circumstances and enhanced data richness [31].

**Table 2:** Overview of the conducted focus groups and interviews

Service provider site	Interview type	Pseudonym	Number of participants (*n* = 24)
1	focus group	FG11	8
2		FG12	6
3		FG13	7
4	interview	I2	1
5		I5	2

### Data collection and analysis

An interview guide for the focus groups was developed by an interprofessional team of physicians and sociologists in consultation with representatives from the participating service providers. The interview guide focused on questions related to the experiences of the relatives.

Preparation and planning of focus groups was conducted between May and July 2014, data collection was carried out from July to November 2014. Focus groups took place in the rooms made available for the purpose by each of the participating service providers. The duration was approximately 100 min. All focus groups and interviews were moderated by the same author (DR) in order to provide a continuous and comparable interview style. In some groups, an additional member of the researcher team was present (KG).

The focus groups were digitally recorded (audio and video), phone-interviews used only digital audio recording. The collected data were transcribed in full text and anonymized. The transcripts were consequently subjected to qualitative content analysis [32]. The software used to support the data analysis was Atlas.ti 7.0.

The decision for using this method of analysis depends not only on the published study protocol [18] but also it is a well-known approach in health care science and often used for interpreting text material. The structured approach of content analysis allows a descriptive view to the experiences of relatives within this kind of care for their patients.

For data analysis the conventional approach to content analysis was chosen [33]. In detail the researchers used a deductive-inductive approach in generating thematic categories. Based on the interview guide, a provisional category system was created initially consisting of attitudes to structural aspects of care such as home treatment and 24-h accessibility and their own experience within the NWpG (deductive approach). This was adapted in the course of the analysis according to the content of the transcripts and was supplemented by emerging new categories (inductive approach) [32].

Transcripts were first coded independently into categories and sub-categories by three different researchers (DR, KG, JV) and then discussed intensively in consensus meetings until agreement was found. However, no inter-rater reliability was determined. Quotations were used to illustrate each of the categories [32]. The same approach to analysis was carried out for both the interviews and the focus groups. Together with a detailed documentation of the research process, the quality principle of intersubjectivity and transparency was achieved [34]. Further quality criteria used were the reflected subjectivity of the researcher and the empirical anchoring of theories developed within the textual data [34].

### Ethics

Ethical approval for this research study was obtained from the ethics committee of the Medical Faculty of the University of Heidelberg in November 2013 (Approval No. S-540/2013). No additional data were evaluated.

## Results

### Sample characteristics

In total, 24 relatives participated to the investigation. Twenty-one relatives took part to the focus groups and three relatives to the interviews. Further details of the number of participants are shown in Table 3. Sixteen were female and eight male. Fifteen relatives were the spouse of a patient. Other relatives’ relationships included parents, siblings, and children. The mean age was 47 years (range between 18 years and 70 years).

**Table 3:** Structural components – main and sub-categories

Main categories	Sub-categories
Home treatment	Openness
	Safety
	Insight in home environment
	Regular contact
24-h accessibility	Safety
	Support
	Protection
	Stability
	Relief
Case-management/relational therapist	Continuity
	Confidential person
	Support
	Expertise
Crisis intervention apartment	

## Key categories

The two key categories discussed in the present study are the structural aspects of the service providers and the experiences of relatives with the NWpG. Main categories are divided into different sub-categories.

## Structural aspects

The structures and facilities offered by all service providers, namely home treatment, 24-h accessibility, case management, relational therapist and crisis intervention apartment are summarized as structural aspects. Table 3 shows the main categories and sub-categories of the structural components common to all participating service providers within the NWpG. Citations will be presented within the text.

### Home treatment

#### Openness

Some meetings with mental health professionals took place at the patient’s home. The relatives reported that the home environment led to more openness from the patients. They preferred the familiar and personal atmosphere rather than the anonymous setting in facilities of the service provider:

*“I saw my husband, he feels much safer and is more open. They tell much more, because the home environment is different than if you are sitting here.” (FG12_R6)*

A high number of participants mentioned the importance of meetings at home, which led to a more relaxed atmosphere between therapist, patient and relative.

#### Safety

The majority of the relatives considered home treatment as a reassurance for themselves as well as for the patients:

*“…that there is someone that comes home. This is a reassurance, I think.” (FG11_R6)*

Seeking help may occur more frequently since the patients do not have to leave their houses and safety zones. This reflects the therapeutic principle of patient-centered care, here realized by meeting the patients in their home and being willing to adapt services according to their changing needs. Home treatment particularly in case of emergency responses was often perceived as an important aspect of safety:

*“Home visits indeed played a major role especially in a crisis - were always great, were always a good support.” (FG13_R3)*

#### Insight in home environment

Appointments in the patient’s home allowed an insight into their private life which might reveal indications for treatment and result in an information gain by the relational therapist. Relatives mentioned that this aspect was lost if the meetings were just held at the facilities of the service providers.

Home treatment was also seen by the relatives as an opportunity to encourage the patient within the home and to motivate them to leave the house. This is especially important for patients with reduced motivation and with the tendency of letting themselves get run down as for example seen with depressive disorders:

*“…if then really also the private environment is involved and the relational therapist knows how they live…that’s again a piece of the puzzle to perhaps be able to better help with…or to be able to support.” (FG11_R7)*

The majority of the relatives approved home treatment but in singular cases it was rejected because it was seen as a violation of privacy:

*“But for me somehow I would not feel so comfortable or rather for me, my home or our home is a place where I relax.” (FG11_R4)*

#### Regular contact

Relatives stated that home treatment with regular contact from the relational therapist could lead to an improved sense of safety not only for relatives but also for patients. Regular appointments that took place independently of acute patient’s needs were regarded as an important beneficial effect from the perspective of relatives:

*“…and it will also be asked regularly how you feel. And there are also appointments…so not just if it is acute, but from time to time in between…for comparison.” (FG11_R3)*

### 24-h accessibility

#### Safety

Most of the service providers implemented a hotline which enabled patients or relatives to contact the health care team around the clock. Many relatives reported that having a direct link to the service provider for any eventuality led to an immense sense of reassurance and relief:

*“To me it is an enormous safety, I have a phone number, so that I could call anytime when I feel overwhelmed, as a catharsis and also if I cannot carry on anymore.” (FG12_R6)*

#### Support

Relatives noticed that in addition to the health and psychological services, support in social care (for example in organizational as well as in financial or legal issues if needed by themselves or the patients) was also available. This support was another component of assistance, which contributed to the relief described by the relatives:

*“There is always someone there and I think that’s a comforting feeling also for my father…not only for us.” (FG12_R1)*

#### Protection

Relatives reported that the 24-h accessibility via the telephone hotline led to an increased sense of protection for themselves as well as for their mentally ill family member. They stated that this 24-h accessibility was an important feature particularly in the case of an emergency:

*“That…before I had to call the police, I can call someone else who still might be able to fix it as a professional.” (FG11_R3)*

#### Stability

Different relatives highlighted the effectiveness and importance of 24-h hotline to the service provider, particularly if immediate support was needed. This easy accessible support around the clock was described as inducing stability:

*“We had one or two times the case that an attack occurred very suddenly…a phone call was sufficient and an hour later support was here. That gave me as a relative a lot of safety and stability.” (FG13_R3)*

#### Relief

Some relatives also mentioned the relief gained by being able to pass on responsibility to the service provider if they could not handle the situation anymore. The availability of this professional support was often mentioned:

*“…one naturally feels then much safer, and of course relieved, it’s not all on oneself and above all, if you experience any problems there is now the possibility for me: I ’ll call the service provider staff, they should solve this. Yes, wonderful.” (FG13_R7)*

### Relational therapist

#### Continuity

The fluctuation in continuity of care among the relational therapists and case managers was a controversial topic among the relatives. Some relatives said that the fluctuation was a big problem especially if the medical or social history of the patient has not been sufficiently handed over and they had to start from the beginning again:

*“…and then there is always a bit of difficulty with the continuity [of the relational therapist]…there will always be fluctuations …but as far as it goes someone should be very careful and make sure that there are not constantly new ones, so that they are not constantly changing.” (FG13_R7)*

On the other hand, there were also positive statements regarding the fluctuation in continuity of care among therapists. It seems that the fluctuation was better accepted if there was transparency and proper information before the change. Some service providers adapted a “tandem-therapist concept”, which means that there were mostly two service provider staff simultaneously at appointments so that the potential substitute knew the patient and his or her history already:

*“…and when there was a representative, then they were informed, competent, so they knew…and that is the most important thing that you can rely on them.” (FG12_R5)*

#### Confidential person

Some relatives described viewing the relational therapist as a friend, who comes to the home for a visit. This was only possible if there was reciprocal trust as well as enough time to establish this level of therapeutic relationship between patient, relative and therapist:

*“…and a lot of personality. Such a personal contact and that as well…yes such a confidentiality was established between [relational therapist] and my husband. Without this fundamental trust he would not open himself. No way…so…and given the fact that there is this good basis of trust…and you have to establish this first…to someone.” (FG12_R6)*

#### Support

Some relatives also reported that patients would establish contact with the service provider in the first instance rather than going to a family member with an issue. A part of their support was thus been taken over by the service provider which led to an immense sense of relief. One relative used the metaphor of the service provider as “anchor” to describe this:

*“I can only agree regarding the support, therefore if he has any problems, he can go to them and does not need to discuss this with me necessarily, because they know him and can assess him very well…and that relieves one of course.” (FG13_R6)*

#### Expertise

The expertise and the professional knowledge were mentioned several times by relatives. This was described as a very important factor for establishing a sense of safety and relief because it filled the relative’s gap in knowledge. The service provider’s know-how was reported to result in a knowledge gain for the relatives, which could lead to new perspectives and an improved ability for the relatives to deal with family members with mental illness.

Another important point reported by relatives was the fact that mental health professionals were not emotionally involved and could be more professional and objective than the relatives in helping the patients. The staff acted as an external and neutral person and had more authority than involved relatives:

*“This trained, good person who knows what he is talking, which we cannot do anymore as an affected person, because we are unable to cope…too many emotions…and I think that is what the service provider brings: time, confidence, and truly with an abundance of professional knowledge.” (FG12_R5)*

### Need for information

Relatives often reported that there is a general lack of information about the modalities of treatment in mental health care. Information was also lacking about the existence of integrated cares services in a community setting for mentally ill persons and the services that they provided. This lack of information led to uncertainty:

*“This is now quite interesting that I do not know exactly how this…what this service is.” (I 5_R1)*

### Requirements

Many relatives emphasized the importance of an individual treatment plan, which was adapted to the needs of the patient. It is well known that empowerment of the patient increases the adherence to the therapy and therefore positively influences the therapeutic outcomes [35, 36]. This is relevant in the treatment of severely mentally ill patients:

*“The care itself relies thank god on the patients, we have found it different in other organizations…without being asked what you actually want, how can I help you…and this is completely different here.” (FG13_R3)*

## Experience

The sub-categories concerning experiences of relatives with care of their family members with mental health problems are relief, support, hand over responsibility, and protection and stability. These four sub-categories will be reported here:

### Relief

Relatives were asked to talk about their experiences with the service provider. One point consistently mentioned was the relief provided. The first experiences were compared to a safety net. Some relatives were looking in vain for support in other institutions and found this trough the integrated care services:

*“The first few days within the service provider were simply, they were just redemption, they were wonderful, it was immediately taken up, they took care of her…she was simply treated humanely.” (FG13_R3)*

### Support

The support experienced trough integrated care services was not just essential for the patients, but was an important source of support for the relatives as well. The metaphor of a heavy basket that they could not hold anymore was used, and that they could hand it over to the service providers and the comfort and help this gave:

*“It’s as said the support. The support to know: There is someone…for him…for him, by whom I’m learning, too.” (FG12_R5)*

### Hand over responsibility

Relatives reported that the mental health service providers were not just working in isolation but in close cooperation with various other therapists and medical practitioners. This led to an experience of patient-centered care, which allowed also for a delegation of responsibility from the relatives, as well as a better flow of information on the patient’s health status:

*“Between the therapist and the service providers there is also a strong connection so far…yes, a good, I do not know what to call it, a good…ring around him, where I think to myself: Okay, I can leave him there.” (FG13_R1)*

### Protection and stability

Relatives said the integrated care service provided protection and stability not just trough the different aspects mentioned above but also through the regular assessment mechanisms. One relative compared this service to the German Association for Technical Inspection i.e., Technischer Überwachungsverein (TÜV), which is a type of regulatory authority. Some relatives reported worries about their own psychological integrity and detrimental effects on their own mental well-being while caring for the patients:

*“I am also of the opinion that actually the diseases are eventually contagious…so, if the partner has somehow certain restlessness or whatever, than that’s somehow contagious now…but for me there is also a certain potential of contagion somehow…[Sounds of agreement from round the group]” (FG13_R5)*

Another central aspect that the relatives mentioned several times was the fact that they didn’t feel being let alone with the patients anymore. The knowledge of integrated care services gave in some cases an increased stability and relief for relatives whether they required the support or not.

*“To have the feeling of not having to stand alone as a relative.” (FG11_A5)*

## Discussion

The purpose of the study was to explore relatives’ experiences within integrated care services for patients with severe mental illness.

The main findings of our study showed that the structural elements such as home-treatment, relational therapist and a 24-h telephone hotline to the mental health care team were valued by relatives of patients with severe mental illness. These structural aspects led to a significant sense of relief for relatives and provided a substantial support in daily life. As a result, an increased feeling of safety and support for relatives was reported as well as a sense of encouragement in not being left alone to deal with a challenging situation.

As illustrated in our main findings, the relational therapist was one of the key-features in reducing carer burden. A well-established long-term connection between therapist and patient through regular contact built an effective therapeutic relationship, which had flow on benefits for relatives in terms of support, information sharing and reduction of responsibility. Similar results have been shown in other studies [35–39].

Therefore, changes in the continuity of care from the relational therapist could present a big challenge for patients and their relatives. This fluctuation cannot be completely prevented and attention should be paid to the organization and planning of such changes in order to minimize stress for patients. This would be even more important when patients experience acute episodes of mental illness because they have fewer resources than when they are stable. An important focus for service providers should be which strategies can be taken to reduce the inconstancy in relational therapist. Some service providers implemented a “tandem-concept” for the relational therapist so that the meetings were always attended simultaneously meaning they could always cover each other, and this continuity was accepted well by both patients and relatives. It could be assumed that personal sympathy may also affect the dynamic o the relational therapist; and that the “tandem concept” could also balance out differences.

It was observed that a consultation with patients and relatives with regard to pending changes resulted in better acceptance of fluctuation in relational therapist and in a lower perceived burden for both patients and relatives.

The results of our qualitative study identified that information provided by the integrated care services was very heterogeneous and not sufficient and satisfactory for most relatives. This lack of information led to an uncertainty about the provided services, which would be crucial for the decision as to whether to participate in such a service.

Additionally, a lack of adequate medical knowledge was described by relatives, which increased the sense of insecurity and perception of high burden of care [40]. Some service providers offered self-help groups for relatives, which were well accepted. Sufficient education for relatives may not only bring advantages for the relative, but may increase the quality of care provided by the relatives [10, 41].

There is much uncertainty regarding the utilization of the services by the relatives for personal questions. Moreover, it was not clear if relatives would access to the integrated care services as they are “just” relatives and not registered in the service provider as are the patients’ they have to care for. In this point further information for relatives is needed.

It is also up for debate whether service providers should address directly to the relatives for recruitment or concerns regarding the patients. The question if and to what extent relatives should be involved in treatment is a point of tension between the autonomy and freedom of choice of the patient and the decision-making ability of patients with a severe mental illness. This should be an important topic for further studies.

Relatives caring for patients with mental health problems may carry a significant risk of being adversely affected in their own psychological health. Previous studies have indicated that effective treatment strategies should address all affected family members caring for patients with chronic and mental illness [42, 43].

### Strengths and weaknesses

The findings of the current study must be viewed under the specific quality criteria for qualitative research. Some limitations have to be considered when interpreting the results. The study was undertaken in five regions of Germany and only included relatives who were interested in taking part in this study, which may have resulted in selection bias of superior motivation within our sample. As usual in qualitative studies, the sample is not intended to achieve representativeness. Moreover, no inter-rater reliability was analyzed to examine the reliability of coding between the three researchers (DR, KG, JV). However, the data supports the importance of integrated care for mental health services in the community setting and contributes to the development of hypotheses for further quantitative research. In addition, lack of specificity of people with different severe mental health problems may bring different needs and burden for the relatives. Representative studies should be used to distinguish the actual needs. Furthermore, we collected no information about the financial situation of relatives. Therefore, a selection bias concerning the 50 Euro reimbursement could not be excluded. Additionally, there is a risk that relatives may have felt under pressure to participate when approached by the organization caring for their relative. However, several relatives mentioned in the focus groups and interviews that they had a high intrinsic motivation to participate. Lastly, an observer bias cannot be ruled out during performing the interviews as well interpreting of data. However, we minimized the observer bias with different strategies as follows: the moderator of interviews (DR) was not completely invited in the aim of the study; open questions were asked during the interviews and focus groups, and the analysis of data was performed by two of three researchers (KG, JV) who were mainly not involved in conducting the study. The results of this qualitative study are not generalizable but are important for the generation of ideas and hypotheses as it is the purpose of qualitative research in general.

## Conclusions

Relatives are an important resource for patients with mental health problems. It can be concluded that relatives also benefit from the services which are offered to patients in integrated care models. These services providers contribute to well-being of relatives reducing burden of care and improve the feeling of safety and security. Moreover, an important need is to ensure a continuity of care for patients and to bridge the gap concerning information needs for relatives. To increase the involvement of relatives in care of mentally ill patients, information about existing mental health services using an integrated care model and their structures should be optimized.
